# Lived Experiences of Turkish Internationally Educated Nurses: A Phenomenological Study

**DOI:** 10.1111/nhs.70046

**Published:** 2025-02-04

**Authors:** Mehmet Gülşen, Dilan Deniz Akan, Salih Tosun

**Affiliations:** ^1^ Department of Medical Services and Techniques İvrindi Health Services Vocational School, Balıkesir University İvrindi Balıkesir Turkey; ^2^ Department of Internal Medicine Nursing Faculty of Health Sciences, Manisa Celal Bayar University Yunusemre Manisa Turkey

**Keywords:** experience, migration, nursing, phenomenology

## Abstract

The international mobility of the nursing workforce is growing. In recent years, Türkiye has seen a notable increase in the number of nurses seeking employment abroad. This study aimed to describe lived experiences of Turkish internationally educated nurses (IENs). A qualitative design with a descriptive phenomenological approach was employed, and the results are reported following the consolidated criteria for reporting qualitative research (COREQ) checklist. Data were collected from September to November 2023 through in‐depth, individual online interviews using a semi‐structured interview form. Sixteen Turkish IENs were interviewed. Through thematic analysis, four themes emerged: push factors of migration, pull factors of migration, positive experiences following migration, and negative experiences following migration. The migration of Turkish nurses to higher‐income countries significantly enhanced their professional and personal lives but also presented considerable challenges. This study underscores the dual nature of migration, offering both opportunities and challenges.

**Summary**

- Migration decisions of Turkish IENs were shaped by push factors such as poor working conditions and economic instability and pull factors such as better career opportunities and higher salaries abroad.
- Turkish IENs experienced positive aspects in their new working environments, such as supportive managers and professional respect, but faced challenges, including language barriers and cultural difficulties.
- The study emphasizes the need for Turkish healthcare authorities to improve nurses' working conditions and highlights the importance of orientation programs for IENs in destination countries.

## 1 Introduction

Migration has continued throughout the history of humanity, from ancient times to the present day, and migration rates have continued to increase steadily over the years (IOM 2024). Globalization, the liberalization of markets, and the increasing demand for healthcare services have contributed to the breakdown of national borders (Toyin‐Thomas et al. 2023). Today, the migration of healthcare professionals, including nurses, has become a trend in Türkiye (Ulupinar, Şen, and Eycan 2024).

Like many other healthcare professionals, nurses have been migrating for more than a century. This migration presents challenges for both origin and destination countries. The negative impact of the international migration of health workers on health systems in origin countries is a worldwide concern (Ung et al. 2024). This concern has encouraged international organizations to act in this regard. In 2010, at the 63rd World Health Assembly, the World Health Organization (WHO) implemented a code of practice to enhance the understanding and ethical management of the process. The Code encourages member countries to share their information on the international recruitment and migration of health personnel (WHO 2010).

The international mobility of the nursing workforce is increasing rapidly (WHO 2020). Nurse migration typically flows from developing to developed countries, driven by aging populations in developed nations, changes in production technology, labor market conditions, and immigration policies (International Labour Organization 2021). Additionally, the availability of better jobs, higher salaries, improved working conditions, superior health infrastructures, clinic or hospital resources, and enhanced education opportunities are among the pull factors for nurse migration (WHO 2020). Pull factors refer to the attractive conditions of the destination country that pull nurses toward migration.

Nurses from countries with lower nurse‐to‐population ratios, like Türkiye, are particularly vulnerable to the push factors that drive this trend (Stokes and Iskander 2021). Push factors refer to the negative conditions in their home country that push nurses to leave. In Türkiye, the nurse‐to‐population ratio is significantly below the Organization for Economic Co‐Operation and Development (OECD) average, with only 2.8 nurses per 1000 people compared to the OECD average of 9.2 (OECD 2024). This shortage is exacerbated by the strong desire of Turkish nurses to work abroad. Surveys reveal that a significant majority of Turkish nurses express a desire to migrate, with 76.3% indicating such intentions according to the Turkish Nurses Association (TNA 2023) and 84.3% in a study by Ulupinar, Şen, and Eycan (2024). These statistics underscore the critical challenge for the Turkish healthcare system in retaining its nursing workforce, which could adversely affect the quality of healthcare in the country. When it comes to internationally trained nurses (IENs), India and the Philippines have been major players in the global market (Thompson and Walton‐Roberts 2019). Türkiye, with its well‐established nursing education system, is now emerging as a new origin country for international nurse migration.

Many different factors lead to international nurse migration. These factors can be categorized as personal, professional, political, and socioeconomic factors (Ung et al. 2024). A quite recent study revealed that concerns about political stability, economic factors, anxieties about the future, a desire for a more prosperous life, and worries about their children's well‐being are pushing healthcare professionals in Türkiye to migrate (Olgay and Yurt 2023). The migration patterns of Turkish nurses align closely with global trends observed in middle‐income countries with comparable socioeconomic dynamics, such as Romania (Druică and Ianole‐Călin 2022), the Philippines (Bretana et al. 2023), and India (Khan et al. 2023). In these countries, common push factors of migration include economic instability, dissatisfaction with workplace conditions, and limited career growth opportunities.

The economic difficulties that emerged after the COVID‐19 pandemic in Türkiye have exacerbated dissatisfaction with wages, as well as with life, among healthcare workers, significantly influencing their intention to migrate (Tosun and Cerev 2023). Over the past decade, the Turkish lira has depreciated substantially against the dollar, while inflation has reached unprecedented levels (Hadi et al. 2023). This combination of economic crises and political instability has created a heightened sense of insecurity among healthcare workers, prompting many to seek more stable opportunities abroad.

Additionally, cultural and societal factors play an important role in shaping the migration decisions of nurses. Cultural challenges, such as the undervaluation of the profession of nursing and the hierarchical structure of the healthcare system, push many nurses to seek work environments abroad where they can experience greater respect and professional autonomy (Yürümezoğlu and Çamveren 2024). Furthermore, in Turkish society, working abroad is often viewed as a prestigious accomplishment that enhances one's economic and social status. Factors such as the higher standards of living in European countries and the presence of relatives who have already migrated encourage nurses to consider migrating (Ultan 2017).

Despite this growing interest in migration, there is a significant research gap regarding the lived experiences of Turkish nurses who have migrated abroad. This study aims to address this gap by exploring the lived experiences of Turkish IENs. Understanding these experiences can provide valuable insights into the challenges they face, the adjustments they make, and the impact of migration on their professional and personal lives. By examining these lived experiences, this study aims to contribute to the literature on international nurse migration and provide practical insights for nursing professionals considering migration.

The research questions of this study were:

- What are the lived experiences of Turkish IENs?
- How do Turkish IENs make sense of these experiences in both personal and professional contexts?

## 2 Research Methodology

### 2.1 Study Design

This qualitative study employed a descriptive phenomenology (transcendental) approach pioneered by Husserl (Polit and Beck 2018). This approach was chosen to explore the lived experiences of Turkish IENs. Phenomenology allows participants to describe how they experience the examined phenomenon (Creswell 2014). The consolidated criteria for reporting qualitative research (COREQ) checklist were used in the reporting of the results of this study (Tong, Sainsbury, and Craig 2007).

### 2.2 Setting and Participants

To explore the experiences of Turkish nurses who migrate to other countries, purposive sampling was used to recruit participants, and snowball sampling was used to enable easy access to those who were eligible. This method allowed the researchers to find participants with diverse characteristics, such as those living in different countries, from different age groups, and with varied experiences. The sample included nurses who had shared their migration experiences on social media platforms, which helped in the recruitment process.

There are no strict rules about sample size in qualitative research. Namey et al. (2016) argued that 8–16 in‐depth interviews are sufficient. In this study, a total of 16 in‐depth interviews were conducted, as data saturation was reached. No one refused to participate or withdrew from the study after the initiation of the data collection process.

Participants were included in the study if they met the following criteria: (1) being of Turkish nationality, (2) having worked as a nurse in Türkiye for at least 6 months, and (3) having worked in a destination country for a minimum of 3 months. These criteria were chosen to ensure that participants had sufficient clinical experience within the Turkish healthcare system to reflect on their migration process meaningfully, as well as adequate exposure to the healthcare system of their destination country to provide insights into adaptation challenges and opportunities. Nurses who had not received their nursing education in Türkiye or who had less than the required work experience were excluded from the study.

### 2.3 Data Collection

A semi‐structured in‐depth interview guide, developed by the researchers based on a comprehensive literature review, guided this study. To ensure the suitability of the interview guide, a qualitative methods expert evaluated and approved the questions. The guide was then pilot‐tested in two interviews. Before the interviews, appointments were scheduled based on the convenience of the nurses and the researchers. No prior relationship with participants was established prior to the commencement of the study. All interviews were conducted via Zoom at the convenience of the participants in their everyday settings from September to November 2023. The duration of the interviews ranged from 40 to 60 min. Each participant was interviewed once.

The first (XX) (male) and second (XX) (female) authors, both nurse academics with PhDs and previous experience in qualitative research methods and interviews, conducted all interviews in Turkish. The choice of conducting interviews in the participants' native language helped foster a comfortable environment, encouraging participants to express their experiences more freely. Prior to each interview, the researchers emphasized the significance of the study and provided an overview of the conditions of the research process. All interviews were audio recorded, and field notes were taken during the interviews. The interview guide comprised five questions on sociodemographic characteristics and four open‐ended questions on the migration experiences of the participants (Table 1). Follow‐up questions such as “How?,” “Why?,” and “Can you explain more about …?” were also asked during the interviews.

**TABLE 1:** The semi‐structured interview guide.

Can you please tell me your reasons for choosing to migrate?
2 Can you please tell me about your initial experiences as a nurse in this new country?
3 Can you please tell me about any challenges you faced in adjusting to the healthcare system here?
4 Can you please tell me whether your expectations about migrating were met?

### 2.4 Data Analysis

For data analysis and interpretation, a thematic analysis method with an inductive approach was used (Sundler et al. 2019). First, to prepare the data for analysis, the audio‐recorded interviews were carefully transcribed verbatim. Second, all the transcriptions were read carefully. Next, initial codes were generated and subsequently grouped under relevant themes. The researchers generated codes only based on the emerging information collected from the participants. Then, each of the initial codes and themes was revisited and refined. Last, the themes were arranged as per the research questions. The third author (XX), in collaboration with the first author (XX), conducted the analyses using the qualitative analysis software MAXQDA Analytics Pro 2024. Thereafter, all authors discussed the analyses and their results until a consensus was reached.

### 2.5 Trustworthiness

Lincoln and Guba (1985) outlined four criteria to ensure trustworthiness: credibility, transferability, dependability, and confirmability. To achieve credibility, the study's results were compared to those in the existing literature. Data collection was continued until the codes started to repeat, indicating data saturation. A significant amount of time was spent with the participants to build trust and gain an in‐depth understanding of their experiences. At the end of each interview, the main points of the interview were briefly summarized for the participants, and any misunderstandings were clarified. To meet the criteria of transferability, a rich description of the setting and participants is provided. The COREQ checklist was utilized to improve the dependability of the study. The confirmability of the results was guaranteed by including direct participant quotes to support the interpretation of the results.

### 2.6 Ethics

Ethical approval was granted by the Local Ethics Committee of Manisa Celal Bayar University, with approval number 20.478.486/1966. Verbal informed consent was obtained from the participants, and their rights to privacy were guaranteed. To protect the privacy of the participants, code names such as P1, P2, … were assigned to them. For confidentiality, the data were kept on a password‐protected flash drive, which was used only by the researchers. The recordings were deleted after the completion of transcription and data analysis.

## 3 Results

Sixteen participants living in three different continents, including 6 men and 10 women aged 26–48, were recruited for the study. The migration destinations of the participants included Germany, the United Kingdom, the United States, Ireland, Australia, Belgium, Sweden, and Switzerland. Five of these participants had master's degrees. Most participants reported increased job and life satisfaction since moving abroad. Additionally, all participants stated that they had no intention of returning to Türkiye. The detailed characteristics of the participants are presented in Table 2.

**TABLE 2:** Detailed characteristics of participants (*N* = 16).

Number	Age	Gender	Marital status	Level of education	Destination country	Total working time (origin/destination)	Economic status following migration	Job satisfaction following migration	Life satisfaction following migration
P1	34	Female	Married	Master's degree	Germany	4.5 years	Improved	Improved	Improved
						0.5 years			
P2	28	Female	Single	Bachelor's degree	Germany	4.5 years	Improved	Improved	Improved
						0.5 years			
P3	26	Male	Single	Bachelor's degree	Germany	1.5 years	Improved	Improved	Worsened
						1.0 years			
P4	41	Female	Married	Master's degree	The United Kingdom	7.0 years	Improved	Improved	Improved
						18.0 years			
P5	31	Female	Married	Master's degree	The United Kingdom	3.0 years	Improved	Improved	Improved
						1.0 years			
P6	42	Male	Married	Master's degree	The United States	11.0 years	Unchanged	Improved	Unchanged
						7.0 years			
P7	30	Female	Single	Bachelor's degree	Ireland	7.0 years	Unchanged	Improved	Unchanged
						1.5 years			
P8	48	Female	Married	Bachelor's degree	The United States	10.0 years	Improved	Improved	Improved
						18.0 years			
P9	30	Male	Single	Bachelor's degree	Australia	3.0 years	Improved	Improved	Improved
						2.0 years			
P10	30	Female	Married	Bachelor's degree	The United States	4.0 years	Improved	Improved	Improved
						1.5 years			
P11	33	Male	Single	Master's degree	Ireland	10.0 years	Improved	Improved	Improved
						1.0 years			
P12	32	Female	Single	Bachelor's degree	Ireland	7.0 years	Improved	Improved	Improved
						1.5 years			
P13	29	Male	Married	Associate degree	Germany	10.0 years	Unchanged	Improved	Improved
						0.5 years			
P14	42	Male	Married	Bachelor's degree	Belgium	10.0 years	Improved	Improved	Improved
						7.0 years			
P15	39	Female	Married	Bachelor's degree	Sweden	5.0 years	Improved	Improved	Improved
						11.0 years			
P16	26	Female	Single	Bachelor's degree	Switzerland	1.0 years	Improved	Improved	Improved
						1.0 years			

**FIGURE 1:**
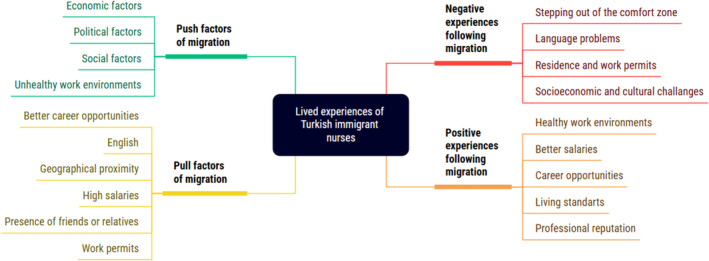
Mind map of the themes and sub‐themes.

Four main themes emerged from the lived experiences of the participants: push factors of migration, pull factors of migration, positive experiences following migration, and negative experiences following migration. Figure 1 presents a mind map of the themes and sub‐themes.

The push factors of migration highlighted the negative conditions in the participants’ country of origin that motivated them to leave. The participants frequently cited financial challenges, including dissatisfaction with low salaries that failed to reflect their professional effort. One participant shared their view, saying, “I was upset because my salary was low. I was doing my job to the best of my ability, but I wasn't making enough money; it wasn't satisfying” (P7). Another explained the disparity they felt, recounting, “One day, the janitor I worked with at the hospital said something that stung. ‘Look,’ she said, ‘I get paid the same as you, sometimes even more.’ It was painful for me to realize that my labor wasn't being financially rewarded” (P12).

Political instability and concerns about justice were also prominent reasons for migration. “I thought that justice was corrupted throughout the country. I didn't think I could have justice if something happened to me. Additionally, since my country received a lot of immigration, I was worried about civil unrest in the future,” expressed one participant (P11). Another pointed to the Gezi Park protests as a turning point, stating, “The Gezi Park protests increased my concerns about the state of democracy and civil liberties in the country” (P6).

Social and workplace‐related factors further compounded the reasons for the participants to leave. The female participants often felt disheartened by societal attitudes toward women and the profession of nursing. “Where we lived was a small town. The way people viewed women and nursing bothered me as a woman,” remarked one participant (P8). Others described their work environment as unsupportive and overly demanding. “The workload was very heavy, and the working hours were very long. Older nurses would often make us do most of the work because they said we were young,” noted one participant (P10). Another recalled, “A workday was 12–14 hours. We were constantly on edge during night shifts. Supervisor nurses were constantly pressuring us” (P3).

On the other hand, the pull factors of migration emphasized the opportunities and advantages of moving abroad. Many participants were drawn by better career opportunities and higher salaries. “Ultimately, the United States is a developed country, with good career opportunities, high living standards, nurses earning better salaries, etc.,” explained one participant (P6). Proficiency in English was also found to be a facilitator. “Since I have an English language education, I wanted to migrate to the English‐speaking United States or United Kingdom,” commented one participant (P4).

Geographical proximity and social connections in destination countries were additional pull factors. “I chose the United Kingdom because it is closer. …because I am the only child of my family, and in case my family needs me, I want to be able to reach them quickly,” shared one participant (P4). Another participant highlighted the emotional comfort provided by people they knew: “Having my cousin here made my decision easier. She helped me settle in and feel less alone” (P16).

The participants reported numerous positive experiences that they had after migrating, which reinforced their decision. Healthy work environments were a recurring theme. “My manager checks in with me every day, asking if I'm okay, if I'm facing any issues, if I need anything, and if I'm adjusting well. Not only is he kind and caring toward me, but he also extends the same kindness and care to everyone,” reflected one participant (P1). Financial stability also improved significantly, as illustrated by another participant: “I am pleased that I receive compensation for every hour I work. While employed in Türkiye in the oncology ward, although my shift officially ended at 4 o'clock, we frequently stayed later due to the extensive handover process, yet there was no payment for this additional time” (P10).

Career development opportunities abroad allowed the participants to grow professionally. “I have advanced in my profession. I am now in a managerial position. I have had the opportunity to participate in many certificate programs, and my hospital covered the expenses. This was unexpected for me,” stated one participant (P4). Higher living standards also enhanced their quality of life. “In contrast to our situation in Türkiye, we were able to easily purchase a car here,” commented another (P1). Moreover, professional respect in their new environments boosted job satisfaction. “My highest level of job satisfaction has been while working here. This is because there is immense respect for my profession, not only within the hospital but also beyond it. For instance, when I mention that I am a nurse, people genuinely show respect,” remarked one participant (P13).

However, migration also presented challenges. Many participants found stepping out of their comfort zones daunting. “There were times at the beginning when I got lost. I didn't know the roads, the streets, the street names… I didn't even have a phone line,” said one participant (P7). Language barriers further complicated their adjustment. “Accent differences make communication even more challenging. In the beginning, you don't understand anyone at your workplace,” explained another (P13).

Bureaucratic processes for obtaining residence and work permits were described as stressful and exhausting. “The processes of obtaining residence and work permits were slow and exhausting,” noted one participant (P3). Additionally, adapting to cultural and lifestyle differences posed challenges. “Adapting to the local food culture here was challenging because the dishes I was used to were not available,” commented one participant (P9).

## 4 Discussion

This study explored the lived experiences of Turkish IENs with the thematic analysis method, revealing four main themes: push factors of migration, pull factors of migration, positive experiences following migration, and negative experiences following migration.

Nurses play a critical role in the healthcare industry. The need for nurses is rising alongside the world's aging population. The International Council of Nurses (2021) estimates that the number of additional nurses needed in the future will reach 13 million. The international mobility of nurses predominantly occurs as a one‐way movement benefiting industrialized countries (Dzinamarira and Musuka 2021; Özaydın, Vural, and Güdük 2024). Based on WHO (2020) data, 15.2% of nurses in high‐income countries are foreign‐born or trained. In line with these statistics, all participants in this study chose developed countries as their migration destinations, further highlighting the tendency of nurse migration toward economically advanced countries. While destination countries gain well‐trained and experienced nurses with little or no expense, nurse migration causes many problems for origin countries due to the loss of qualified workforce (Moshiri, Mohammadi, and Yarahmadi 2022). Within this framework, the WHO global code of practice advises that the international recruitment of health personnel should be conducted in accordance with the principles of transparency, fairness, and promotion of the sustainability of health systems in developing countries (WHO 2010).

In this study, participants highlighted various push factors driving Turkish nurses to seek opportunities outside Türkiye, including dissatisfaction with salaries, concerns about civil rights and freedoms, gender inequality, safety issues, and heavy workloads. These push factors align with previous studies, which have consistently reported similar concerns among Turkish nurses considering migration (Ulupinar, Şen, and Eycan 2024; Olgay and Yurt 2023). These problems are linked to larger issues in Türkiye's healthcare system, which has been under growing pressure in recent years. One major reason is the large number of migrants and refugees in the country. According to the International Organization for Migration (IOM 2024), Türkiye currently hosts 3.9 million migrants, 90% of whom are Syrians fleeing war, making it one of the countries hosting the largest numbers of foreigners seeking international protection. This influx has significantly increased the demand for healthcare services, placing additional burdens on an already strained system. With one of the lowest nurse‐to‐population ratios among OECD countries, Turkish nurses face heavier workloads and longer working hours. Besides, the political instability in the country after the 2016 coup attempt (Alpdoğan 2023) has added to the stress and insecurity faced by healthcare workers. Wanting to move to another country can be seen as either a sign of dissatisfaction with one's current life or simply a desire to seek better opportunities. Many studies indicated that job satisfaction among nurses in Türkiye was moderate to low (Gulsen and Ozmen 2020; Soysal, Menekşe, and Kıraç 2022). It is believed that the recent depreciation of the Turkish lira and the subsequent decrease in purchasing power have further exacerbated the dissatisfaction among Turkish nurses, driving them to seek better financial stability and professional fulfillment abroad. This economic instability, combined with unhealthy working environments, has contributed significantly to the decision of many nurses to migrate.

Pull factors, such as better career opportunities, higher salaries, and an improved quality of life, attract Turkish nurses to countries perceived as offering greater professional and personal fulfillment. Similarly, Boboc, Ghiță, and Vasile (2024) found that Romanian nurses were influenced by opportunities for employment and career advancement, as well as better working conditions, when deciding to migrate. According to Olgay and Yurt (2023), Turkish healthcare workers in the United Kingdom experienced significantly improved working conditions and higher salaries, which enhanced their overall job satisfaction and quality of life. Besides, proficiency in the English language is a significant facilitator of migration, particularly to English‐speaking countries like the United States and the United Kingdom. In this context, the review by Cubelo et al. (2024a) further emphasized the significance of reassessing language prerequisites and providing individualized support to address challenges, thereby enhancing job satisfaction and retention among IENs.

The participants reported both positive and negative aspects of their new environments. Similarly, a recent study on the professional experiences of Turkish nurses who migrated to Germany revealed a mix of positive and negative experiences. The study revealed that better earnings compared to their home country and flexibility in working hours positively impacted the nurses. However, IENs also faced challenges such as high cultural diversity, communication barriers, racism, an inadequate number of nurses, and a heavy workload (Sezer 2023). In line with these findings, a study on nurse migration in Australia, Germany, and the UK revealed that IENs often experienced discrimination and poorer career opportunities, emphasizing the need for structured integration programs to support their transition (Smith et al. 2022).

The positive aspects of the current work environments of the participants were highlighted, including the caring and supportive nature of their managers, the respect they received for their days off, and receiving payment for every hour they worked. The statements of the participants indicated that they felt more valued in their new work environments, which was reflected in their job satisfaction. This aligns with the findings of previous studies emphasizing the critical role of supportive work environments in enhancing nurse retention and job satisfaction (Rothbart et al. 2024).

In contrast, negative experiences following migration, such as the challenges of obtaining residence and work permits, language barriers, and feelings of loneliness and isolation, were also brought to light. The difficulties of adapting to a new cultural and social environment, as well as the absence of familiar foods, posed significant challenges for the participants. The process of migration involves stepping out of one's comfort zone in various aspects and can be mentally and physically draining. In their systematic review, Rajpoot et al. (2024) identified several challenges faced by IENs during migration, including communication and language problems, culture shock, differences in clinical practices and professional development, and issues related to discrimination and racism. Although the Turkish IENs who participated in this study did not report challenges in having their nursing qualifications recognized, IENs also experience complicated pathways for their qualifications to be recognized in their destination country. The absence of a clear and structured recognition process may result in deskilling and limit their ability to practice effectively (Cubelo et al. 2024b). As noted by Cubelo et al. (2024b), the licensure pathway for IENs requires collaboration among various stakeholders, with the acknowledgment of their prior education and clinical experience being critical to facilitating their transition to registered nursing roles. To enhance the readiness of IENs for effective healthcare delivery, Cubelo, Parviainen, and Kohanová (2024c) also recommended that nursing policy and practice integrate language instruction, cultural sensitivity training, and customized educational approaches into bridging programs.

### 4.1 Limitations

The main limitation of this study was that the experiences and recommendations shared by the participants were based on their perceptions and interpretations and may not encompass the full spectrum of challenges and recommendations relevant to all IENs. Furthermore, the sample consisted only of nurses who migrated to certain countries and did not include the experiences of nurses who migrated to other countries. Future research could explore the experiences of nurses from diverse cultural backgrounds who have immigrated to a wider range of countries. Additionally, conducting longitudinal studies to track the long‐term experiences and integration of IENs in their new work environments would provide valuable information about the evolving challenges and successes that nurses encounter over time.

## 5 Conclusions

In conclusion, while the migration of Turkish nurses to higher‐income countries has brought about significant improvements in their professional and personal lives, it also presented considerable challenges. The findings of this study highlighted the dual nature of migration, offering both opportunities and challenges. By understanding the factors that drive the migration of nurses and the conditions that support their retention, policymakers can develop more effective strategies to manage the global nursing workforce. The results of the study emphasized the critical need for Turkish healthcare authorities to develop strategies to retain nurses and improve their working conditions within Türkiye. Moreover, the results demonstrated the importance of supportive work environments and orientation programs in retaining IENs in destination countries. Future research should explore longitudinal experiences of IENs to understand their integration and long‐term career trajectories. Furthermore, cross‐country comparisons could provide a broader understanding of migration dynamics and policy interventions.

### 5.1 Relevance for Clinical Practice

Understanding the push and pull factors of migration influencing nurse migration can inform strategies aimed at improving nurse retention in origin countries, such as Türkiye. The reported challenges faced by IENs, including cultural adaptation and communication barriers, emphasize the need for targeted orientation programs. These programs should include mentoring, clinical orientation, and workshops to familiarize nurses with local health policies and practices, ensuring safer and higher‐quality care. Clinical orientations for IENs should differ from those provided to local nurses, focusing on gradual and progressive learning. Culturally sensitive mentors and native counterparts from similar backgrounds can help IENs adapt more easily by providing guidance, reducing unfamiliarity, and fostering supportive relationships. Such initiatives can facilitate smoother transitions for IENs, helping them integrate into new healthcare systems and improving their overall well‐being.

## Author Contributions

**Mehmet Gülşen:** conceptualization, writing – original draft, investigation, methodology, validation, writing – review and editing, formal analysis, project administration, data curation, supervision. **Dilan Deniz Akan:** conceptualization; investigation; writing – original draft; methodology; validation; project administration; data curation. **Salih Tosun:** data curation, formal analysis, methodology, visualization.

## Ethics Statement

Ethical approval was granted by the Local Ethics Committee of Manisa Celal Bayar University, with approval number 20.478.486/1966.

## Conflicts of Interest

The authors declare no conflicts of interest.

## Data Availability

Research data are not shared.
